# Sphingolipids produced by gut bacteria enter host metabolic pathways impacting ceramide levels

**DOI:** 10.1038/s41467-020-16274-w

**Published:** 2020-05-18

**Authors:** Elizabeth L. Johnson, Stacey L. Heaver, Jillian L. Waters, Benjamin I. Kim, Alexis Bretin, Andrew L. Goodman, Andrew T. Gewirtz, Tilla S. Worgall, Ruth E. Ley

**Affiliations:** 1 Department of Microbiome Science, Max Planck Institute for Developmental Biology, Tübingen, 72076 Germany; 2 Department of Pathology and Cell Biology, Columbia University, New York, NY 10032 USA; 3 Center for Inflammation, Immunity, and Infection, Institute for Biomedical Sciences, Georgia State University, Atlanta, GA 30303 USA; 4 Department of Microbial Pathogenesis, Yale University School of Medicine, New Haven, CT 06520 USA

**Keywords:** Symbiosis, Bacterial genes

## Abstract

Gut microbes are linked to host metabolism, but specific mechanisms remain to be uncovered. Ceramides, a type of sphingolipid (SL), have been implicated in the development of a range of metabolic disorders from insulin resistance (IR) to hepatic steatosis. SLs are obtained from the diet and generated by de novo synthesis in mammalian tissues. Another potential, but unexplored, source of mammalian SLs is production by Bacteroidetes, the dominant phylum of the gut microbiome. Genomes of *Bacteroides* spp. and their relatives encode serine palmitoyltransfease (SPT), allowing them to produce SLs. Here, we explore the contribution of SL-production by gut *Bacteroides* to host SL homeostasis. In human cell culture, bacterial SLs are processed by host SL-metabolic pathways. In mouse models, *Bacteroides*-derived lipids transfer to host epithelial tissue and the hepatic portal vein. Administration of *B. thetaiotaomicron* to mice, but not an SPT-deficient strain, reduces de novo SL production and increases liver ceramides. These results indicate that gut-derived bacterial SLs affect host lipid metabolism.

## Abstract (web summary)

Ceramides are a type of sphingolipid (SL) that have been shown to play a role in several metabolic disorders. Here, the authors investigate the effect of SL-production by gut *Bacteroides* on host SL homeostasis and show that microbiome-derived SLs enter host circulation and alter ceramide production.

## Introduction

Sphingolipids (SLs) are important structural and bioactive signaling molecules in mammals, with significant roles in the development of metabolic disorders^1–3^. Ceramides, a subtype of SL, are the best-studied SL in insulin resistance (IR)^4–6^. In animal models, reduction of ceramide (d18:1/16:0) in the liver alleviates IR^2,7,8^, and pharmacological inhibition of ceramide-related pathways in the intestine leads to improved glucose homeostasis^9^. In human populations, several studies have associated levels of hepatic or plasma ceramides to IR^10–12^. Regulation of SL levels across tissues is dependent on the inputs from multiple signaling pathways and is tightly controlled by de novo synthesis, recycling, and intestinal uptake of SLs^6,13^. Dietary SLs are a significant source of exogenous SLs to the host and can impact cholesterol absorption^14^, hepatic lipid accumulation^14^, obesity^15^, and insulin sensitivity^16^. Part of the effects of dietary SLs on metabolic disorders can be attributed to the ability of these lipids to alter hepatic ceramide levels^14^.

SLs are also produced by gut bacteria of the phylum Bacteroidetes^17^, including common genera (i.e., *Bacteroides*, *Prevotella* and *Porphyromonas*), that on average constitute ~30-40% of the human gut microbiome^17^. These prominent members of the gut microbiome have the necessary enzyme serine palmitoyltransferase (SPT), which is responsible for the first step of de novo SL synthesis. *Bacteroides* produce both odd-chain length sphinganine (Sa - d17:0)^18^ as well as even-chain length Sa (d18:0), which are similar or identical to mammalian even-chain Sa (d18:0)^19,20^. Bacteria-derived SLs have been shown to signal into inflammation-related pathways in the colon^21,22^ but nothing is known about their ability to affect pathways involved in host lipid metabolism.

Here, we investigate the effects of *Bacteroides*-derived SLs on host hepatic SL metabolism. Using cell culture assays, we show that bacterial-like SLs are taken up and processed in mammalian SL metabolic pathways. When monocolonized in germfree mice, lipids originating from *Bacteroides thetaiotaomicron (B. theta)* cells can be visualized in mouse gut epithelial tissue. Compared with monocolonizatoin with an SPT-deficient *B. theta* strain, association with WT *B. theta* led to higher hepatic SLs. In conventionally raised mice fed diets devoid of fat, oral supplementation of WT *B. theta* reduced de novo ceramide production rates compared with the SPT-mutant, which is expected if *B. theta*’s SL production supplements the mouse diet. Moreover, in mice with diet-induced IR, administration of WT *B. theta* supplementation led to higher hepatic ceramide levels compared with the SPT-mutant. Together, these findings indicate that *Bacteroides*-SLs produced in the gut provide an endogenous source of SLs that impacts host lipid homeostasis.

## Results

### Human epithelial cells uptake and process bacterial SLs

**Fig. 1:**
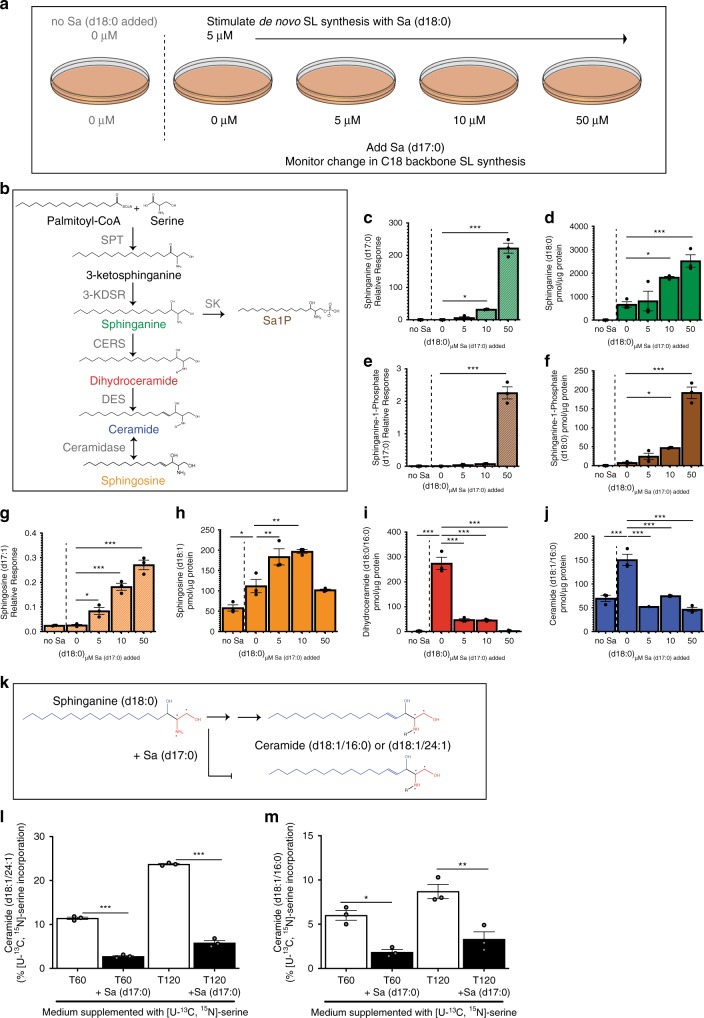
Odd chain SLs produced by gut microbes can inhibit host de novo synthesis of even chain SLs. **a** Illustration of experimental setup where Caco-2 cells were stimulated with 5 μM of Sa (d18:0) and challenged with increasing concentrations of Sa (d17:0). **b** SL de novo synthesis pathway. SPT (serine palmitoyltransferase), 3-KDSR (3-keto-dihydrosphignosine reductase), CERS (ceramide synthase), DES (desaturase), SK (sphingosine kinase). **c**–**j** SL synthesis was induced in proliferating Caco-2 cells with addition of 5 μM Sa (d18:0), then cells were dosed with increasing concentrations (5, 10, 50 μM) of Sa (d17:0) to monitor the ability of Sa (d17:0) to inhibit flux of C18-base length SLs through the SL synthesis pathway. Labels and graph colors refer back to the corresponding metabolite in (**b**); graphs of metabolites with C18 sphingoid backbones have solid bars while graphs of metabolites with C17 sphingoid backbones are represented by striped bars. Cells were harvested 1 h after addition of lipids. Means ± standard error of the mean (SEM) of three biological replicate experiments (*n* = 3) (one-way ANOVA comparing concentrations to 0 μM Sa (d17:0) added, *p < 0.05, **p < 0.01, ***p < 0.001), are plotted for (**c**) Sa (d17:0), (**d**) Sa (d18:0), (**e**) sphinganine-1-phosphate (Sa1P) (d17:0), (**f**) Sa1P (d18:0), (**g**) So (d17:1), and (**h**) So (d18:1) (**i**) dihydroceramide (d18:0/16:0), (**j**) ceramide (d18:1/16:0). **c**–**j**, **k** Diagram of incorporation of [U–^13^C,^15^N]-serine (red) to identify newly synthesized SLs. [U–^13^C,^15^N]-serine was added to culture medium and [M + 3] isotopes due to [U–^13^C,^15^N]-serine incorporation into newly synthesized SLs were monitored by LC-MS. Sa (d17:0) was added to determine if this metabolite decreased the de novo synthesis of SLs with a C18 length sphingoid base. Percent incorporation of [U–^13^C,^15^N]-serine was measured for (**l**) ceramide (d18:1/16:0) and (**m**) ceramide (d18:1/24:1) after 60 (T60) and 120 min from the addition of the [U–^13^C,^15^N]-serine. Bars represent the mean ± SEM of three biological replicates (two-sided *t*-test, *p < 0.05, **p < 0.01, ***p < 0.001). Source data are provided as a source data file. For all figures with bars, height of bar = mean and error bar is SEM.

To investigate the potential for bacterial SLs to influence host hepatic SL homeostasis, we first wanted to establish whether human epithelial cells could take up and process bacterial SLs. We supplemented Caco-2 cells with Sa (d18:0) to stimulate de novo SL synthesis, then dosed cells with different amounts of Sa (d17:0) (Fig. 1a). The difference in the chain length of Sa produced by bacteria (d17:0) and host cells (d18:0) allows their discrimination in culture. We observed that Caco-2 cells took up Sa (d17:0) within 1 h of addition to the medium (Fig. 1c). Sa (d18:0) levels increased (Fig. 1d), indicating that addition of Sa (d17:0) led to decreased flux of Sa (d18:0) through the synthesis pathway. Increases in sphinganine-1-phosphate (d17:0) levels (Fig. 1e) were commensurate with levels of added Sa (d17:0), showing that sphinganine kinase acted on the bacterial-like Sa. We also observed increases in sphinganine-1-phosphate (d18:0), which could be a mechanism for the cell to handle the build-up of Sa (d18:0) (Fig. 1f).

Sa (d17:0) was incorporated into complex SLs through the de novo synthesis pathway as Sa (d17:0) was converted to So (d17:1) (Fig. 1g). So (d18:1) levels rose with the addition of Sa up to a point and then the synthesis of So (d18:1) declined (Fig. 1h). In addition, we observed decreased levels of dihydroceramide (d18:0/16:0) (Fig. 1i) and ceramide (d18:1/16:0) (Fig. 1j), suggesting an overall inhibition of de novo C18 long chain base ceramide production (also observed at lower concentrations; Supplementary Fig. 1A, B). These results show that while the shorter bacterial-like Sa (d17:0) was processed by the SL metabolic pathways, the processing of Sa (d18:0) was reduced and resulted in the accumulation of Sa (d18:0).

### Bacterial SLs inhibit the processing of mammalian SLs

**Fig. 2:**
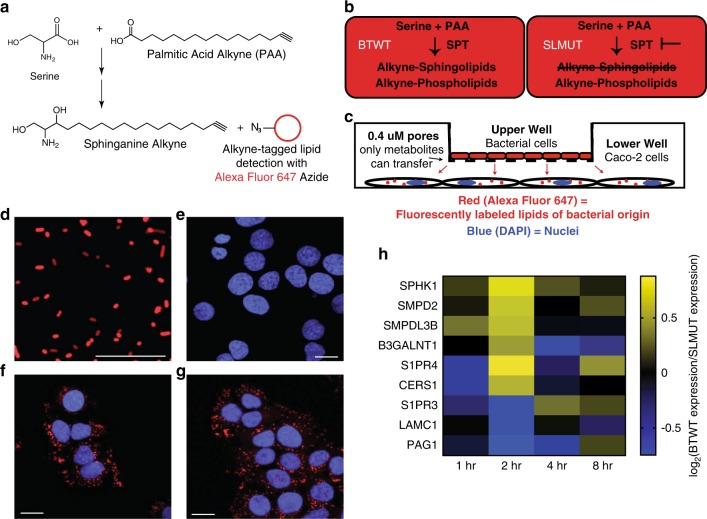
SL production by BTWT regulates SL metabolism genes. **a** Incorporation of palmitic acid alkyne (PAA) into complex SLs allows fluorescent detection of bacterially-derived lipids using an azide (N_3_) conjugated fluorophore (Alexa Fluor 647). **b** Metabolism of PAA in BTWT vs. SLMUT. SPT = serine palmitoyltransferase. **c** Illustration of the transwell coculture system, showing transfer of PAA derivatives from bacterial cells in upper well to Caco-2 cells in lower well. **d** Confocal image showing alkyne-tagged lipids detected in BTWT cells grown with PAA (red - Alexa Fluor 647; detection by click chemistry). Scale bar is 20 μm. Confocal microscopy image of Caco-2 cells in the lower transwell after 6-h exposure to (**e**) cell culture media (**f**) BTWT-PAA or (**G**) SLMUT-PAA. **e**–**g** Alkyne-tagged lipids were detected by Alexa Fluor 647 (red) and DNA was stained using DAPI (blue). Images are representative of three independent experiments. Scale bar is 20 μm. **f** Heatmap showing the average time-zero normalized log_2_ change in gene expression between BTWT and SLMUT in transwell with Caco-2 cells for two biological replicate 8-h time course experiments (yellow = higher, blue = lower for log_2_ BTWT/SLMUT relative expression). Source data are provided as a source data file.

To further test the effects of bacterial SLs on host de novo SL synthesis, we incubated cells with [U–^13^C,^15^N]-labeled serine to monitor newly synthesized SLs. A reduction in the amount of newly synthesized SLs would indicate that Sa (d17:0) inhibits the synthesis of C18 base SLs (see schematic in Fig. 1k). We detected the incorporation of [U–^13^C,^15^N]-serine into newly synthesized ceramide (d18:1/24:1) and ceramide (d18:1/16:0). At the end of the two-hour incubation in medium supplemented with isotopically-labeled serine, SL pools were composed of ~24% newly synthesized ceramide (d18:1/24:1) (Fig. 2l) and ~9% newly synthesized ceramide (d18:1/16:0) (Fig. 2m). However, when the medium was supplemented with Sa (d17:0), SL pools were only composed of ~6% newly synthesized ceramide (d18:1/24:1) (Fig. 2l) and ~3% of newly synthesized ceramide (d18:1/16:0) (Fig. 2m). These observations indicate that the addition of Sa (d17:0) to Caco-2 cells inhibited flux through the de novo synthesis pathway of C18-base SLs. Together, these results indicate that (i) Sa (d17:0) can be taken up by human gut epithelial cells, then (ii) metabolized by mammalian SL-processing pathways, while (iii) inhibiting the production of mammalian C18-base SLs.

### Lipids transfer between bacterial and Caco-2 cells

Brown et al.^22^ recently showed that deletion of SPT in *Bacteroides thetaiotaomicron* VPI 5482 completely ablated SL production. Here, we used *B. thetaiotaomicron* (BTWT) and a strain with an inactivated SPT that also showed ablation of SL production (SLMUT), in both whole cells and OMVs^23,24^ (Supplementary Fig. 2; see Methods), to assess how Caco-2 cells responded to whole bacterial cells with and without SLs. BTWT and SLMUT were both grown in medium supplemented with palmitic acid alkyne (PAA; Fig. 2a). We used PAA, a precursor for SL synthesis, because it allowed labeling of SLs in *B.thetaiotaomicron* without causing toxicity. Although both strains can incorporate PAA into complex lipids, effects of lipid transfer seen in the BTWT condition are specific for the ability to synthesize SLs (Fig. 2b). *B. thetaiotaomicron* strains were placed in a transwell system with Caco-2 cells (Fig. 2c). For both strains, alkyne-labeled lipids transferred to Caco-2 cells within 4 h (Fig. 2f–g).

### Caco-2 cell responses to *B. theta* and SPT-deficient mutant

To assess the impact of lipid transfer on the recipient cells, we compared the transcriptomes of Caco-2 cells exposed to either BTWT or SLMUT cells in a transwell system. Genes involved in SL metabolism were differentially regulated over an 8-hour period depending on the treatment (targeted analysis of 107 SL-related genes, Supplementary Data 1, Fig. 2h, Supplementary Data 2). For instance, the sphingosine-1-phosphate receptor gene S1PR4 was upregulated early in Caco-2 cells co-incubated with BTWT compared with SLMUT. S1PR4 serves as a receptor for gut microbiome produced N-acyl amides, and has been suggested to respond to microbiome-derived SLs^25^. Additional genes encoding enzymes in the SL-processing pathway, and upregulated by Caco-2 cells in response to BTWT compared with SLMUT, included a sphingosine kinase (SPHK1), sphingomyelinases (SMPD2, SMPDL3B), and a ceramide synthase (CERS1). SPHK1 is involved in the phosphorylation of the long chain base sphingosine to sphingosine-1-phosphate, and SMPD2 and SMPDL3B in the hydrolysis of the phospholipid sphingomyelin into ceramide and phosphocholine^3^. CERS1 produces ceramides with a C18:0 acyl chain length shown to influence obesity-related IR in skeletal muscle^26^. Untargeted gene expression analysis comparing BTWT to SLMUT exposed Caco-2 cells revealed clusters of genes with similar expression patterns that may have important but undefined roles in SL-dependent host-microbe interactions (Supplementary Fig. 3, Supplementary Table 1). These results indicate that the SL-production capacity of bacterial cells shapes the gene expression response of Caco-2 cells in this in vitro system, in a manner consistent with the sensing and uptake of SLs.

### Bacterial SLs transfer into mouse intestinal epithelium

**Fig. 3:**
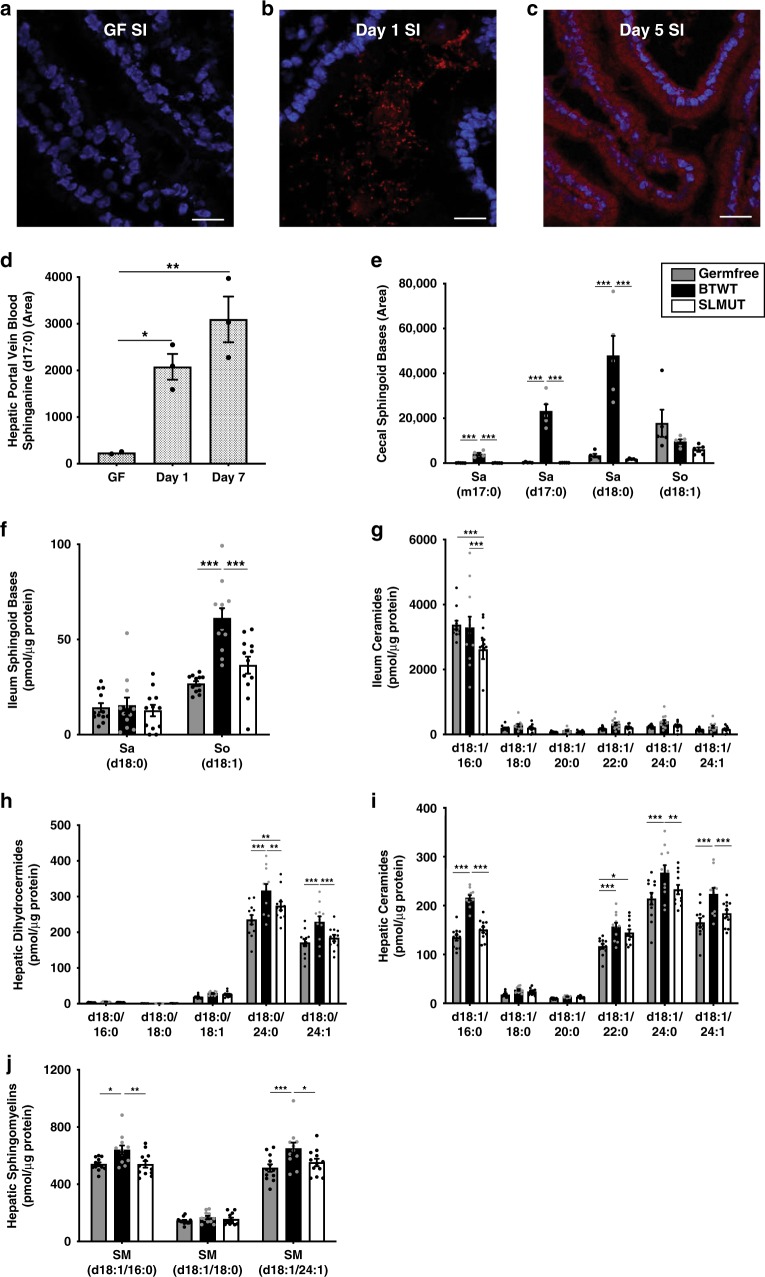
Bacterial SLs are uptaken in GI tract and portal vein and affect tissue SLs. Confocal microscopy of **a** small intestine (SI) tissue of germfree mice, **b** SI tissue of germfree mice inoculated with BTWT grown in PAA (BTWT-PAA) 3 h after oral gavage, and **c** SI tissue of germfree mice after 5 days of daily gavage with BTWT-PAA. PAA metabolites were detected with Alexa Fluor 647 azide (red) using click chemistry, and nuclei of the intestinal epithelial cells were stained using DAPI (blue). Scale bar is 20 µm. Representative images of four mice. **d** Sphinganine (d17:0) levels in acid base-treated hepatic portal vein blood of germfree mice gavaged daily with BTWT. For hepatic portal vein blood samples, means ± SEM of LC-MS measurements are plotted for: GF = germfree (*n* = 2), one day of daily gavage, Day 1 (*n* = 3); 7 days of daily gavage, Day 7 (*n* = 3) (one-way ANOVA, Tukey’s multiple comparison test, *p < 0.05, **p < 0.01). **e** Cecal long chain base SLs. Bar charts represent SL abundance ± SEM for five mice per condition. (two-way ANOVA, Tukey’s multiple comparison test, ***p < 0.001). **f**–**j** Bar charts represent mean SL abundance ± SEM for: GF (gray, *n* = 12 mice), monoassociation with BTWT (black, *n* = 11 mice) or monoassociation with SLMUT (white, *n* = 12 mice); two-way ANOVA, Tukey’s multiple comparison test, *p < 0.05, **p < 0.01, ***p < 0.001). **f** Ileal long-chain sphingoid base SLs; **g** Ileal ceramides; **h** Hepatic dihydroceramides; **i** Hepatic ceramides; **j** Hepatic sphingomyelins. Source data are provided as a source data file. For all figures with bars, height of bar = mean and error bar is SEM.

We next wished to observe the direct transfer of lipids from bacterial cells to host cells in vivo. BTWT grown in PAA supplemented media (BTWT-PAA) was administered by oral gavage to 5-week old germfree (GF) Swiss-Webster (SW) mice. PAA-metabolites, readily observed in bacterial cells (Fig. 2d), transferred from the lumen into host gut epithelial tissue (Fig. 3a–c). However, although SLs are likely included in the PAA-labeled pool, other metabolites were likely included. To assess more specifically if bacterial SLs transited into host tissue distal to the intestine, we introduced BTWT cells containing Sa (d17:0) and Sa (d18:0) in nearly equivalent amounts (Supplementary Fig. 4) via daily oral gavage to germfree SW mice over a one-week period, and measured SLs in host tissue over the course of the week. On days 1 and 7, we measured higher Sa (d17:0) in hepatic portal vein blood in BTWT-treated compared with untreated mice (Fig. 3d). These results establish that *Bacteroides*-SLs can transit from bacterial cells to the gut epithelium and hepatic portal vein.

### Hepatic SLs in germfree and conventionalized mice

Having found that *Bacteroides*-derived lipids can move into host gut tissue, we next sought to characterize their impact on host hepatic SL levels. SL metabolism of germfree mice has been shown to differ from that of colonized mice and is normalized when they are colonized with a standard microbiome^27^. We confirmed that (i) GF mice have higher hepatic ceramides compared with conventionally raised mice, and that (ii) conventionalization of GF mice by inoculation with a complex microbiota normalizes hepatic ceramides^28,29^ (Supplementary Fig. 5). But whether SL synthesis by members of the Bacteroidetes in the gut microbiome is responsible for changes in hepatic SLs is unclear.

### SL-production capacity of *B. theta* affects liver SLs

To tightly modulate the SL-production of the microbiome, we monocolonized 4-week-old GF SW mice with BTWT or SLMUT. Both colonized the mouse cecum at comparable levels (Supplementary Fig. 6A). After 6 weeks, levels of three long chain base SLs synthesized by BTWT, Sa (d17:0), 15-methylhexadeca Sa (m17:0), and Sa (d18:0), were significantly higher in the ceca of BTWT-colonized mice compared with SLMUT- and GF-mice (Fig. 3e). In contrast, levels of So (d18:1), which is not synthesized by BTWT, was similar between treatments (Fig. 3f). Cecal dihydroceramides and ceramides with longer acyl chains (C22:0, C24:0, C24:1) were elevated in SLMUT-mice, but cecal sphingomyelins were similar between conditions (Supplementary Figs. 6B–D). Thus, as recently described^22^, the SL-production capacity of gut microbes affects the levels of SLs in the cecum.

Consistent with uptake and desaturation of SL bases (d18:0) from the lumen, levels of the unsaturated long chain base So (d18:1) were highest in the ileum of BTWT-mice (Fig. 3f). Dihydroceramide (d18:0/18:1), and dihydroceramide (d18:0/24:0) (Supplementary Fig. 6E) were also significantly elevated in the ileum of BTWT-mice compared with SLMUT- and GF- mice. Additionally, ileal ceramide levels (d18:1/18:0, d18:1/20:0, d18:1/22:0, d18:1/24:0, d18:1/24:1) trended higher in the BTWT-mice compared with SLMUT and GF mice (Fig. 3g), while ceramide and sphingomyelin (d18:1/16:0) were higher in ilea of the GF- and BTWT-mice (Supplementary Fig. 6F). These observations suggest that SL long chain bases (Sa (d18:0) and Sa (d17:0)) are taken up from the lumen by intestinal epithelial cells and metabolized through de novo SL synthesis pathways. It is also possible that host sensing of these bacterial products could induce changes in host SL metabolism.

The difference in SL levels between BTWT and SLMUT treatments that we observed in the gut lumen and intestine were also evident in the liver. Hepatic dihydroceramide and ceramide levels were significantly higher in the BTWT- compared with SLMUT-mice for several prominent ceramide species. For instance, the hepatic levels of dihydroceramide (d18:0/24:1) (Fig. 3H), ceramide (d18:1/16:0), ceramide (d18:1/24:0), ceramide (d18:1/24:1) (Fig. 3i), sphingomyelin (d18:1/16:0), and sphingomyelin (d18:1/24:1) (Fig. 3j) were individually higher in BTWT compared with SLMUT and GF conditions. Hepatic sphingosine was elevated in the BTWT- and SLMUT-mice compared with GF-mice (Supplementary Fig. 6G). These results indicate that SL production by gut* Bacteroides* can affect levels of liver ceramides.

### *B. theta* reduces hepatic SL de novo production rate

**Fig. 4:**
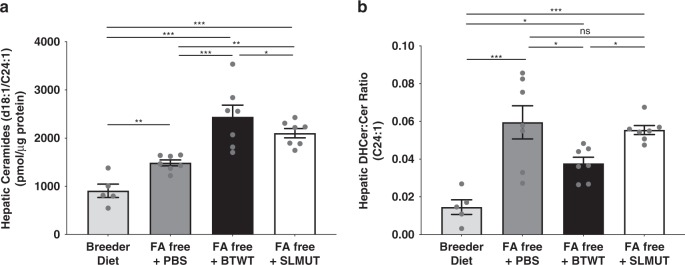
Oral supplementation of BTWT inhibits hepatic de novo SL synthesis in vivo. **a** Hepatic ceramides in mice on a breeder diet or a fatty acid free (FA free) diet that stimulates hepatic de novo SL synthesis. Mice on the FA free diet were gavaged with PBS (control), BTWT, or SLMUT. **b** Hepatic dihydroceramide (DHCer) to ceramide (Cer) ratios for ceramide (d18:1/24:1). Mean values ± SEM are plotted, *n* = 7 per condition, (one-way ANOVA, Holm-Sidak multiple comparison test, *p ≤ 0.05, **p < 0.01, ***p < 0.001, ns = not-significant). Source data are provided as a source data file. For all figures with bars, height of bar = mean and error bar is SEM.

If the gut microbiome is supplying SLs to the liver, or the host senses higher levels of SL, rates of de novo SL synthesis could be affected. To assess this possibility, we placed mice (5-week-old female SW) on a diet (fatty-acid free, or FAF) known to increase hepatic lipogenesis^30^, then orally administered BTWT, SLMUT or vehicle control for 2 days. As expected, after 3 weeks on the FAF diet, hepatic de novo SL synthesis was elevated compared with controls (breeder chow; Supplementary Fig. 7). BTWT treatment was associated with the highest hepatic ceramide levels (Fig. 4a). The ratios of dihydroceramide to ceramide indicated reduced synthesis of the most abundant ceramide (d18:1/24:1) in the BTWT compared with the SLMUT and vehicle controls (Fig. 4b). These results indicate that when mice are fed a SL-poor diet, gut bacteria can act as an endogenous source of lipids, or somehow signal in such a way that decreases the rate at which SLs are newly synthesized.

### *B. theta* and SPT-mutant affect liver ceramides in IR model

**Fig. 5:**
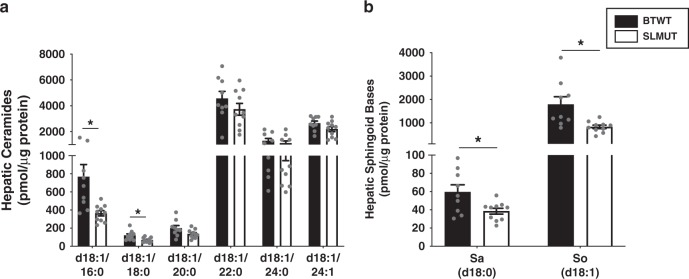
Increased hepatic ceramides in mice supplemented with BTWT as compared with SLMUT. Bar charts represent mean SL abundance ± SEM for (**a**) Hepatic ceramides and (**b**) hepatic sphingoid bases of BTWT treated (black bars) and SLMUT treated (white bars) mice. **a**, **b** Observations with significant differences in SL abundance are marked with stars SLMUT (two-sided *t*-test, *p < 0.05). For BTWT *n* = 9 and for SLMUT *n* = 10. Source data are provided as a source data file. For all figures with bars, height of bar = mean and error bar is SEM.

Incorporation of bacterial SL into host ceramide metabolism can be envisaged to result in broad influence on host metabolism. For example, elevated hepatic ceramide levels have the potential to affect insulin sensitivity in the host in mouse^1,2,7,8^ and human^10^ studies. Hence, we hypothesized that BTWT may contribute to elevated liver ceramides during obesogenic conditions related to IR. We chose to explore this possibility using a well-established mouse model of IR: C57BL/6J mice fed a high-fat diet. We orally gavaged conventionally raised C57BL/6J mice with BTWT or SLMUT (in this instance, an SPT knock-out strain, see Methods) for 7 days, at which time they were switched to a high-fat diet with daily gavage for 7 days and maintained them on the high-fat diet for 21 days thereafter. Mice were then switched to back to a chow diet for 9 days to reduce any effects of the high-fat diet itself while receiving daily gavage of bacterial cells. Consistent with our previous observations, the BTWT-mice had higher hepatic levels of ceramide (d18:1/16:0) and ceramide (d18:1/18:0) (Fig. 5a), sphinganine (d18:0), sphingosine (d18:1) (Fig. 5b) and dihydroceramide (d18:0/18:1) (Supplementary Fig. 8a) relative to the SLMUT-mice; sphingomyelins were equivalent (Supplementary Fig. 8b). These results are consistent with findings that oral administration of the SL-producers *Prevotella copri*^31^ and *Bacteroides fragilis*^32^ increases IR in mice. More generally, these findings support the notion that bacterial-derived SLs can impact host metabolism.

## Discussion

Together, the results of this study point to a potential role for SL production by *Bacteroides* spp. to modulate the levels of bioactive lipids in the liver. We show that gut epithelial cells sense and respond to the presence of bacterial SLs, and that these SLs can be processed via mammalian SL pathways. To achieve these effects, gut-derived SLs may not travel to the liver and affect SL pools directly. Indeed, although we could detect bacterial (C17) SLs in the portal vein of mice, these were undetectable in the liver. This implies that in the host, bacterial SL may be degraded and the fatty acid recycled. Our results do show that mice adjust hepatic de novo production of SLs in response to the SL-production capacity of gut bacteria, however, indicating that the gut bacteria can enhance the pool of SL available to the host. Gut bacteria thus constitute an endogenous source of SLs that, similar to diet-derived SLs, can supply SLs to the host and influence hepatic SL pools^14^.

Several large metagenomics-based gut microbiome studies in humans have linked elevated levels of gut Bacteroidetes to IR^31–34^. For instance, in a study of 292 Danes, Le Chatelier et al.^35^ noted a reduction of *Bacteroides* in low-richness gut microbiomes associated with hosts with lower IR. In a study designed to disentangle metformin treatment from T2D using 784 metagenomes, Forslund et al.^36^ report the Bacteroidetes genus *Paraprevotella* as one of the six genera enriched in untreated T2D. Pederson et al.^31^ linked IR and elevated Bacteroidetes in the gut of 277 non-diabetic individuals. Several mechanisms have been put forward to explain the link between Bacteroidetes and IR, such as via the metabolism of bile acids^33,37^ and FXR signaling^8,32^. That host SLs levels are linked to IR^9,32^, and that Bacteroidetes produce SL, suggests that *Bacteroides* SL may provide a mechanistic link between the gut microbiome and host SL homeostasis, with potential influence on IR.

The gut microbiome influences host metabolism through mechanisms that include inflammation^38^, energy metabolism^39^, and the synthesis of bioactive molecules^40^ that interact with host metabolic pathways. Although the potential pool of metabolism-regulating metabolites generated by the gut microbiota is large, only a few microbial metabolites with defined roles in affecting host metabolism These metabolites include short chain fatty acids^41^, secondary bile acids^42^, endotoxin^43^, trimethylamine^44^, and indoles^45^. Bioactive lipids produced by the gut microbiota have the potential to pass the epithelial barrier and interact with host metabolism, and this class of microbial metabolites, including the SLs, remains to be further explored for their roles in human metabolism.

## Methods

### Caco-2 cell culture

Human epithelial colorectal adenocarcinoma cells (Caco-2, ATCC) were cultured in Dulbecco’s modified Eagle’s medium (DMEM, Thermo Fisher Scientific) supplemented with 10% fetal bovine serum (FBS, Gibco). Plates were seeded at 5 × 10^5^ cells per 10 cm plate, incubated at 37 °C with 5% CO_2_, and experiments took place 48 h after the initial seeding.

For lipid competition experiments, sphinganine (d18:0) (Avanti Polar Lipids) was added to the medium at a concentration of 5 µM. Sphinganine (d17:0) (Avanti Polar Lipids) was also added to plates at concentrations of 0, 5, 10, and 50 µM. The lipid competition experiment was done in triplicate and repeated in triplicate using a lower stimulating concentration of sphinganine (d18:0) of 1 µM with the addition of sphinganine (d17:0) at concentrations of 0.5, 1, 2, and 10 µM. Plates in which there was a media change but no addition of lipid were also collected. Cell pellets from plates were collected after a 1-h incubation and stored at −80 °C.

We used [U–^13^C,^15^N]-serine to monitor de novo SL synthesis in Caco-2 cells. To metabolically label newly synthesized SLs, Caco-2 cells were grown in 10% FBS in DMEM supplemented with a stable isotope of serine [U–^13^C (99%),^15^N (99%)]-serine (Cambridge Isotopes) at a concentration of 2.2 mM. Medium supplemented with [U–^13^C,^15^N]-serine was added to the cells at the same time as 5 µM of sphinganine (d17:0) and the abundance of newly synthesized SLs was quantified at 60 and 120 minutes after the medium change. Sa (d17:0) exposed cells were compared with cells that were supplemented with [U–^13^C,^15^N]-serine but had no additional lipid addition. Newly synthesized SLs were identified by quantifying peaks of [M + 3] labeled SLs with the same chromatography properties as the unlabeled SL.

### Bacterial culturing

*Bacteroides thetaiotaomicron* strain VPI 5482 (“*B. theta*” wild type, BTWT) and the SLMUT strains are from a previously generated transposon insert library^23^. Specifically, SLMUT consists of BTWT with a transposon insertion in the gene BT0870, which is annotated to have 8-amino-7-oxononanoate synthase activity and is homologous to a gene with serine palmitoyl transferase (SPT) activity in *Bacteroides fragilis*^24^ and the known SPT in yeast. The insertion is in a position 88% from the start of the gene. Unless otherwise stated, BTWT and SLMUT strains used in experiments described below were grown under anaerobic conditions at 37 °C in either chopped meat broth (ThermoFisher Scientific/Remel), supplemented brain heart infusion media (BHIS), or a minimal medium with glucose as the sole carbon source (MMG)^46^. The minimal medium consisted of, per L: 13.6 g KH_2_PO_4_, 0.875 g NaCl, 1.125 g (NH_4_)_2_SO_4_, 5 g glucose, (pH to 7.2 with concentrated NaOH), 1 mL hemin solution (500 mg dissolved in 10 mL of 1 M NaOH then diluted to final volume of 500 mL with water), 1 mL MgCl_2_ (0.1 M in water), 1 mL FeSO_4 _× 7H_2_O (1 mg per 10 mL of water), 1 mL vitamin K3 (1 mg/mL in absolute ethanol), 1 mL CaCl_2_ (0.8% w/v), 250 µL vitamin B12 solution (0.02 mg/mL).

### BT0870 knockout strain (Δ0870-SLMUT)

To generate an in-frame deletion of BT0870 in *B. thetaiotaomicron*, the strain *B. thetaiotaomicron* VPI-5482 *tdk* was used^47^. Specifically, two 800–1000 bp regions, each flanking the gene to be deleted, were PCR-amplified (NEB Q5 Hot Start High-Fidelity DNA Polymerase) and cloned into EcoRV-HF and NotI-HF linearized pExchange_tdk. Strains and primers are listed in Supplementary Table 2. The assembled construct was transformed into *E. coli* S17-1 λpir (Biomedal), plated on LB agar-streptomycin-carbenicillin plates, and transformants screened for incorporation of the plasmid. Final concentrations of antibiotics and selection agents were as follows: erythromycin 25 μg/ml, gentamicin 200 μg/ml, streptomycin 100 μg/ml, carbenicillin 100 μg/ml, FUdR 200 μg/ml. To conjugate cells, recipient and donor cells were inoculated from overnight cultures (*B. thetaiotaomicron tdk* at 1:1000; *E. coli* transformant at 1:250) and grown to early exponential phase (OD600 0.2-0.3), when the donor and recipient strains were combined in a 1:1 ratio and centrifuged for 20 min at 4000 RPM at room temperature. The bacterial pellet was resuspended in 100 μl BHIS, plated as a puddle on BHIS-10% defibrinated sheep blood agar plates, and incubated aerobically at 37 °C for 20 h. The conjugation puddle was then scraped, serially diluted in PBS, and incubated aerobically at 37 °C on BHIS-10% defibrinated sheep blood-gentamicin-erythromycin agar plates. Colonies were screened for merodiploids via PCR, cultured overnight in liquid BHIS, and serially diluted onto BHIS-10% defibrinated sheep blood-gentamicin-FUdR agar plates. Colonies were PCR screened for deletion of the gene and confirmed via Sanger sequencing. This strain is differentiated from the transposon insertion mutant (SLMUT) as Δ0870-SLMUT and was used in the high-fat diet feeding experiments (below).

### Thin layer chromatography (TLC)

To resolve SL species, lipid extracts were spotted on non-fluorescent 20 × 20 cm glass backed silica plates (Millipore Sigma) and separated using a 65:25:4 Chloroform: Methanol: Ammonium Hydroxide solvent system for 25 min. A standard composed of sphingosine (d18:1), ceramide (d18:1/12:0), and sphingomyelin (d18:1/12:0) (Avanti Polar Lipids) was used to identify spots. All plates were developed in an iodine chamber overnight, with the exception of FS2C, which was imaged with primuline (0.01 mg/mL in 4:1 v/v acetone:dH_2_O) under trans-UV.

### Inhibition of SL synthesis in BTWT

BTWT and SLMUT were grown in BHIS supplemented with 1 µM of myriocin (Sigma-Aldrich), an inhibitor of SPT, to inhibit de novo SL synthesis. Lack of SL species in SLMUT was confirmed by both liquid chromatography-mass spectrometry (LC-MS) and TLC analysis of lipid extracts (Supplementary Figs. 2A–C).

### Outer membrane vesicle (OMV) preparation

BTWT and SLMUT cultures were separated into fractions (whole cell, cell membranes, outer membrane vehicles (OMVs)) and SL composition was evaluated by LC-MS. To separate the cell fraction (for whole cells and membranes) from the supernatant fraction (for OMVs), 100 mL of 18-h bacterial cultures grown at 37 °C in minimal media were spun at 3220 × *g* for 20 min, the supernatant was moved to a clean tube, and the spin repeated. To prepare OMVs, the supernatant was filtered twice using a 0.22 µm pore membrane (Corning Inc.). 60 mL of each culture was spun at 140,000 × *g* for 2 h at 4 °C. OMV pellets were washed in PBS and spun at 140,000 × *g* for 2 h at 4 °C, then resuspended in 100 µL PBS and stored at −80 °C prior to lipid extraction. Purity of the OMV fraction was confirmed by negatively staining the OMV preparation and imaging by transmission electron microscopy at the Cornell Center for Materials Research at Cornell University. For membrane extraction, half of each bacterial pellet was resuspended in 10 mL membrane extraction buffer (MEB; 50 mM Tris-HCl, 150 mM NaCl, 50 mM MgCl_2_, pH 8.0). The suspension was sonicated in 2 × 30 s intervals, then spun 500 × *g* for 10 min at 4 °C. The supernatant was spun at 140,000 × *g* for 2 h at 4 °C, then the membrane fraction pellet was washed in PBS and respun at 140,000 × *g* for 2 h at 4 °C. Pellets were resuspended in 1 mL PBS and stored at −80 °C prior to lipid extraction^48^. Lipids were extracted from each fraction using the method of Bligh and Dyer^49^ and acid-base treated as described below (see SL measures: acid base hydrolysis). Lipid films were resuspended in 1:1 dichloromethane:methanol prior to analysis by LC-MS.

### Caco-2 transwell incubation with *B. thetaiotaomicron* strains

To assess the effects of metabolite transfer from BTWT to intestinal epithelial cells, Caco-2 cells were incubated with BTWT and SLMUT in tissue culture dishes (Corning Inc.), wherein bacterial cells were placed on a 0.4 µM filter situated 1 mm above the monolayer of Caco-2 cells. 50 mL cultures (OD_600_ of 0.25) grown in minimal medium supplemented with 25 µM palmitic acid alkyne (PAA, Cayman Chemical) were washed three times in PBS and resuspended in 6 mL of 10% FBS in DMEM. One milliliter of bacterial suspension was added to upper-well inserts of the 6-well transwell culture dished. Contents of the upper bacterial insert and the lower Caco-2 cell filled plate were collected 4 h after the addition of the bacteria. Alkyne-containing metabolites in bacterial and Caco-2 cells (plated on UV-sterilized #1.5 glass coverslips in 6-well plates) were labeled with Alexa Fluor 647 azide using the Click iT cell reaction buffer kit (Thermo Fisher Scientific). Caco-2 cells that were not grown in transwell with bacterial cells/alkyne-containing metabolites were also incubated with the Click reaction buffer containing Alexa Fluor 647 to control for non-specific fluorescence. Bacterial cells were mounted onto glass slides and imaged on a LSM 710 confocal microscope (Zeiss) using Zeiss Zen software (Zeiss Zen 2012 SP1black edition).

### RNA-seq of Caco-2 cells

RNA was isolated from Caco-2 cells incubated in transwell plates with BTWT or SLMUT cultures using the Trizol reagent (ThermoFisher) according to manufacturer’s instructions. Samples were collected from BTWT and SLMUT conditions over an 8-hour time period (0, 1, 2, 4, 8 h) and the time course was performed twice. The NEB Next Ultra RNA library kit for Illumina (NEB) was used to prepare sequencing libraries, which were sequenced using 50 bp single end reads on the Illumina HiSeq 2500 platform. The 18 libraries (duplicates of BTWT and SLMUT 1, 2, 4, and 8-h timepoints (16 libraries) in addition to duplicates of the time 0 timepoint (two libraries)) were multiplexed using Illumina barcodes and read across two lanes of a flow cell (nine libraries per lane) for a total of 474,824,584 reads and an average of 26,379,143 reads per sample. Reads were mapped to the human genome using the STAR aligner^50^ with an average of 85% (range 80–87%) of total sequenced reads mapping to the transcriptome. Whole transcriptome analysis of differential expression patterns over the time course was done using the maSigPro package implemented in R^51^. Analysis of the expression of SL processing genes was done with a set of 107 manually curated genes from gene ontology categories involved in SL, ceramide, sphingosine, or sphingomyelin metabolism and are detailed in Supplementary Data 1. For heatmaps, expression values were averaged across replicates and genes with a greater than twofold change in normalized expression values are visualized in Fig. 2h.

### Animal experiments

All experiments involving animals were performed according to Protocol #2010-0065 approved by the Cornell University Institutional Animal Care and Use Committee. Mice were maintained on a 12-h light/dark cycle and housed at constant temperature and humidity. All gavages of bacterial cultures were prepared from overnight cultures. Cells were centrifuged, washed and resuspended in sterile PBS and administered in a volume of 0.2 mL at a concentration of 10^8^ CFU/mL unless otherwise noted.

### Transfer of labeled bacterial lipids to mouse epithelial cells

Five to six-week-old female germfree Swiss Webster (GF SW) (Taconic Biosciences) were purchased and shipped to Cornell University, where they were allowed to acclimate for 48 h in their shipper. Upon removal from the shipper, mice were either immediately sacrificed or gavaged with overnight cultures of BTWT grown in MMG supplemented with 25 µM PAA. Mice were then housed 3–4 mice per cage and fed an autoclaved breeder diet (5021 LabDiet) ad libitum. Mice were gavaged daily with the same dose of PAA-labeled BTWT for 4 additional days until sacrifice. On the day of sacrifice, mice were fasted for 6 h then gavaged with PAA-labeled BTWT (as above) 1 h before sacrifice. Intestinal tissue was harvested for confocal imaging as described below.

Alkyne containing metabolites in bacterial cells (plated on UV-sterilized #1.5 glass coverslips in 6-well plates) were labeled with Alexa Fluor 647 using the Click iT cell reaction buffer kit (Thermo Fisher Scientific). Bacterial cells were mounted onto glass slides and imaged on an LSM 710 confocal microscope (Zeiss).

At the time of sacrifice, small intestinal tissue was cut into three equal sections. Then, 4 cm of tissue from the duodenum, jejunum, ileum, in addition to the whole length of the colon were embedded in O.C.T media and snap-frozen using isopentane and liquid N_2_. Blocks were cryosectioned into 8 µm sections, set and fixed on a glass slide using 4% paraformaldehyde. Slides were stained using the Click iT cell reaction buffer kit (ThermoFisher Scientific) and imaged on an LSM 710 confocal microscope (Zeiss) at the Cornell University Biotechnology Resource Center.

### Hepatic portal vein blood SLs

To assess intestinal SL uptake, 5-week-GF SW mice (Taconic Biosciences) were allowed to acclimate for 2 days in their germfree shipping container. After this acclimation period, mice were either immediately sacrificed or gavaged with BTWT cultures grown overnight in MMG. Mice were housed in sterile filter top plastic cages under specific pathogen free (SPF) conditions and sacrificed 6 h post-gavage on day 1 and 7 of the experiment. Blood was collected from the hepatic portal vein by euthanizing the mice using CO_2_ followed by cervical dislocation. Heparin (100 µL–30 IU/mL, Sigma) was added to the cavity before nicking the hepatic portal vein and blood was collected using a Pasteur pipet. Hepatic portal vein blood was frozen in liquid N_2_ and stored at −80 °C.

### Monoassociation of GF mice with BTWT or SLMUT

GF SW mice were bred in-house and caged in rigid sterile isolators. Mice (females, 3–4- week old) were transferred to flexible bubble isolators and inoculated with either BTWT or SLMUT by oral gavage. Mice were housed 3–4 per cage, fed an autoclaved breeder diet (5021, LabDiet) ad libitum and sterility was checked biweekly. Mice were sacrificed 6 weeks after inoculation. After decapitation, livers, PBS flushed ileum tissue, and cecal contents were collected, flash frozen in liquid N_2_ and stored at −80 °C until processed for SL analysis. Colonization efficiency was monitored by determining the colony forming units per gram (CFU/g) of cecal content of sacrificed mice. 50 mg of cecal content per sample (seven samples per condition) was weighted and added to 1 mL of BHIS. Serial dilutions (1:10) of the slurry were made to the dilution 1:10^8^. Serial dilutions were plated on BHIS agar plates and incubated at 37 °C in an anaerobic chamber overnight before counting colonies.

### Mice used in hepatic SL profiling

Germfree: GF SW mice (female) were bred in-house and caged in rigid sterile isolators. GF mice were sacrificed at 5 weeks of age. Conventionalized mice: 5-week-old female GF SW mice were inoculated with 0.2 mL of fecal slurry made from three mouse pellets homogenized in 3 mL of sterile PBS. Pellets were obtained from SPF Swiss Webster mice housed in the conventional corridor of the Cornell Mouse Facility. Conventionalized mice were housed in sterile filter top cages and sacrificed a week after colonization. Conventionally raised: 5-week old conventionally raised female mice were obtained from litters two generations after breeding pairs from the germfree colony had been conventionalized.

For all mice: after euthanasia by decapitation, livers were collected, flash frozen in liquid N_2_ and stored at −80 °C until processed for SL analysis.

### Induction of hepatic de novo SL synthesis

Five-week-old female SW mice (Taconic Biosciences) were fed either a breeder (5021 LabDiet: calories from protein—23%, carbohydrates—53%, fat—24%) or a fatty acid free diet (TD.03314 Teklad: calories from protein—24%, carbohydrates—76%, fat—0%) ad libitum for 21 days while housed in SPF conditions with 2–5 mice per cage. Mice were gavaged with BTWT or SLMUT 24 h before sacrifice, and then again prior to fasting (6 h before sacrifice). After decapitation, livers were collected, flash frozen in liquid N_2_ and stored at −80 °C until processed for SL analysis.

### Insulin-resistant mice administered BTWT or SLMUT

Seven-week-old male C57BL/6J mice (Jackson Laboratory) on a chow diet (Diet 5001, LabDiet) were gavaged with BTWT or SLMUT for 7 days and then placed on a high-fat diet (HFD; D12492, Research diet, Inc.). Mice were then gavaged for 7 additional days with BTWT and SLMUT and kept on the HFD for an additional 21 days. When mice were then switched back to a chow diet and gavaged daily with BTWT or SLMUT for 9 days before sacrifice. All gavages were 0.2 mL of cultures of BTWT or Δ0870-SLMUT (10^8^ CFU/mL) grown in BHIS and washed in PBS. Mice were euthanized by cervical dislocation and livers were frozen in liquid N_2_ until processed for SL analysis.

### Lipid extractions

Liver, ileum, and colon tissue were all homogenized in PBS using tubes with sterile 1 mm zirconium beads (OPS diagnostics) in a bead beater homogenizer (BioSpec products) for 2 min. Intestinal tissue was placed on ice and then subjected to another round of bead beating to ensure homogeneity of the sample. Protein concentrations of homogenates were measured using a Lowry protein assay (BioRad) and equal concentrations of samples were loaded on to 96-well plates for lipid extractions. Liver samples were extracted in 1:1 dichoromethane:methanol according to the details below with 400–800 µg of protein per sample while colon and ileum samples were extracted with 100–200 µg of protein per sample. Hepatic portal vein whole blood (150 µL) was loaded on to 96-well plates for lipid extraction.

Cecal tissues were weighed before lipids were extracted according to the Folch method^52^ in 4 mL of 2:1 chloroform:methanol. After 2 h of constant vortexing on a plate vortexer, 800 µL of 0.9% sodium chloride in water solution was added to the extraction. Samples were briefly vortexed again before spinning samples at 2000 × *g* for 15 min to separate the aqueous and organic phases. The lower organic phase was then transferred to a new tube where 800 µL of 0.9 % sodium chloride was added in order to ensure an organic phase free of cecal debris. These extractions were spun again at 2000 × *g* for 15 min and the organic phase was transferred to a glass tube and dried under nitrogen gas. The remaining lipids were weighed and resuspended at an equal concentration for subsequent mass spectrometry analysis.

Cell pellets were resuspended in 100 µL of PBS and protein concentrations were assessed using the Lowry method. Equal amounts of cell suspension as measured by protein concentration (100–150 µg) were loaded onto 96-well plates for SL extractions. Bacterial cell pellets were washed and resuspended in PBS and protein concentrations were assessed using the Lowry method. Equal amounts of cell suspension as measured by protein concentration were loaded onto 96-well plates for SL extractions.

All samples loaded onto 96-well plates had 50 µL of 1 µM C12 ceramide (d18:1/12:0) (Avanti Polar Lipids) added as an internal standard and 50 µL of 10% diethylamide diluted in methanol. For the lipid extraction, 450 µL of 1:1 dichloromethane:methanol was added to each sample and vortexed overnight on a plate shaker. After the overnight incubation, an additional 900 µL of 1:1 dichloromethane:methanol was added to the samples and incubated on a plate rotator for an additional hour before spinning samples at 2000 × *g* for 15 min to separate cell debris from the lipid extracts. The supernatant was transferred to a new 96-well plate for analysis by mass spectrometry as detailed below.

### SL measures: acid base hydrolysis

Blood samples and microbial cultures were broken down to their SL long chain base backbones using harsh acid and base treatment to obtain estimates of total SL levels using the methods outlined in ref. ^53^. In brief, equal volume of hepatic portal vein blood (150 µL per experiment) or protein normalized microbial cultures were added to 500 µL of methanol supplemented with 4 µM of 1-deoxysphinganine-D3 (C_18_H_36_D_3_NO) (Avanti Polar Lipids) and vortexed on a plate vortexer for 1 h. Samples were then spun down at 21,130 × *g* in a microcentrifuge for 5 min to remove cell debris. Supernatants were transferred to polypropylene tubes and 75 µL of concentrated hydrochloric acid was added before incubating the samples overnight (16–20 h) at 65 °C. After overnight incubation, concentrated potassium chloride (10 M) was added to samples and a lipid extraction using chloroform was used to extract hydrolyzed SLs. The organic phase of the lipid extraction was dried under nitrogen gas and resuspended in 200 µL of 1:1 dichloromethane:methanol before adding C12 ceramide (d18:1/12:0) (Avanti Polar Lipids) internal standard to samples on a 96-well plate and analyzing SL levels by mass spectrometry as detailed below.

### Lipidomic profiling

SL abundance was measured by liquid chromatography-mass spectrometry (LC-MS) using methods modified from Bui et al.^54^. Specifically, 4 µl of lipid extract from each sample was injected into an Agilent 1200 HPLC (Agilent Poroshell 120 column) linked to an Agilent 6430 triple quadrupole mass spectrometer. Mobile phase A consisted of methanol/water/chloroform/formic acid (55:40:5:0.4 v/v); Mobile phase B consists of methanol/acetonitrile/chloroform/formic acid (48:48:4:0.4 v/v). After pre-equilibration for 6 sec, the gradient gradually increases to 60% mobile phase B and 100% mobile phase B that is held for 1.9 min. Flow rate was 0.6 mL/min. The duration of a run was 9.65 min. Ions were fragmented using electrospray ionization in positive mode and selective reaction monitoring (SRM) allowed for the detection of SL specific transitions. The method was validated for SLs listed in Supplementary Table 3. Peak calls and abundance calculations were done using MassHunter Workstation Software Version B.06.00 SP01/Build 6.0.388.1 (Agilent). Final concentrations of samples were calculated from a standard curve for each SL (Supplementary Table 3). For Sa (d17:0) base metabolites, no standard curve was available and the response of the mass spectrometer normalized to the standard (Ceramide (d18:1/12:0)) was used for abundance calculations.

### Statistical analyses

All data are represented as the mean ± SEM unless otherwise noted. Statistical tests are denoted in figure legends and were implemented using Prism 8.3.0 (GraphPad) or using the Tukey C, nlme, and multcomp packages implemented in R.

### Reporting summary

Further information on research design is available in the Nature Research Reporting Summary linked to this article.

## Supplementary information

Supplementary information

Description of Additional Supplementary Files

Supplementary Data 1

Supplementary Data 2

Reporting Summary

## Data Availability

Data for Fig. 2h and Supplementary Fig. 3 have been deposited in the Sequence Read Archive under the accession number PRJNA613677. Data underlying Figs. 1–5 and Supplementary Figs. 1, 2, 4, 8 are provided as Source Data files. All other data are available as supplementary materials or from the corresponding author upon request.

## Source data

Source data
