# Supplementary material for: Sphingolipids produced by gut bacteria enter host metabolic pathways impacting ceramide levels
Source: Nat Commun. 2020 May 18;11:2471. doi: 10.1038/s41467-020-16274-w (PMC7235224; doi:10.1038/s41467-020-16274-w)
Supplement: Supplementary file 1 — Supplementary information [file 41467_2020_16274_MOESM1_ESM.docx]

Supplementary Materials for

**Sphingolipids produced by gut bacteria enter host metabolic pathways impacting ceramide levels**

Johnson et al.

**This PDF file includes:**

Supplementary Figures 1 - 8

Supplementary Tables 1- 3

**Supplemental Figures**

**Supplementary Figure 1. Effects of Sphinganine (d17:0) on Sphinganine (d18:0) supplemented cells.**

(A - B) SL synthesis was induced in proliferating Caco-2 cells with addition of 1 μM Sa (d18:0), then cells were dosed with increasing concentrations (1, 2, 10 μM) of Sa (d17:0) to monitor the ability of Sa (d17:0) to inhibit flux of C18-base length SLs through the SL synthesis pathway. Cells were harvested 1 hour after addition of lipids. Means ± SEM of 3 biological replicate experiments (n=3) are plotted for (A)

dihydroceramide (d18:0/16:0) and (B) ceramide (d18:1/16:0).

(C) Chromatograms of Sa (d17:0) after addition of 0, 5, 10, 50 μM of Sa (d17:0) to Caco-2 cells. Source data are provided as a source data file.


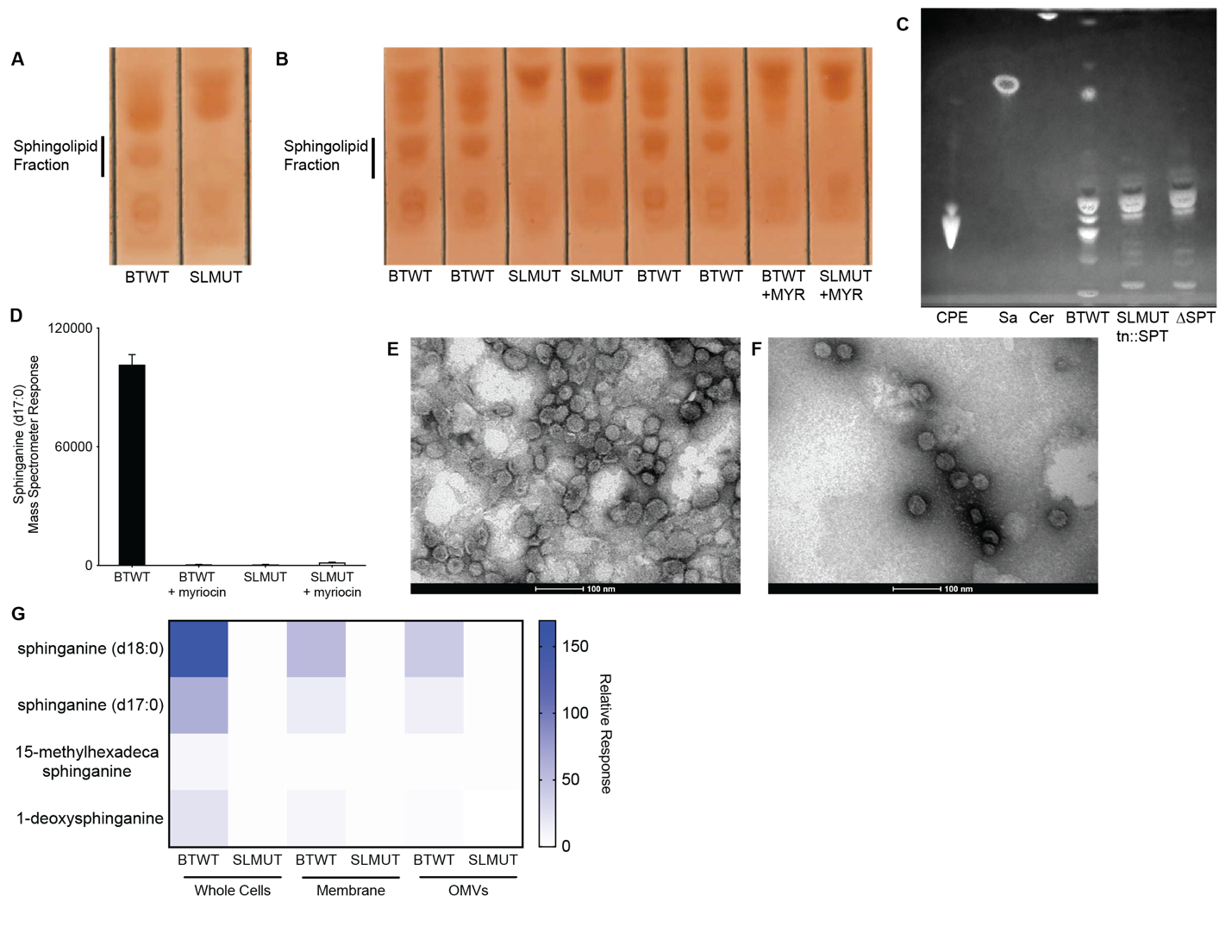


**Supplementary Figure 2. Genetic disruption of the putative serine palmitoyl transferase (SPT) gene by a transposon or drug treatment with the SPT inhibitor myriocin (myr) both inhibit sphingolipid synthesis in *Bacteroides thetaiotaomicron*.**

(A) Thin layer chromatography (TLC) of BTWT and SLMUT lipid extracts showing the SL fraction.

(B) TLC of lipid extracts showing the SL fraction. Shown are BTWT (4 lanes), SLMUT (2 lanes), and each strain treated with myriocin (+ MYR; 2 lanes for BTWT and 1 lane for SLMUT), a chemical inhibitor of sphingolipid synthesis.

(C) TLC of BTWT, SLMUT, and ∆0870-SLMUT strains.

(D) Mass spectrometry measurements of the major sphingolipid, sphinganine (d17:0), in BTWT and SLMUT strains treated or not treated with myriocin. Experiments were performed in duplicate and profiles represent measurements from one representative experiment.

(E-F) Electron micrographs of OMVs fractionated from BTWT (D), and SLMUT (E) cultures. Purity of OMVs was confirmed by the presence of 20 – 250 nm sized particles from log phase cultures. Scale bar is 100 nm.

(G) LC-MS based measurement of four sphingolipids identified in BTWT but absent in SLMUT for whole cells, membrane fractions, and isolated OMVs. Source data are provided as a source data file.

TLC images (A-C) are representative of three independent experiments and electron micrographs of OMV isolations (E-F) are representative of two independent experiments.


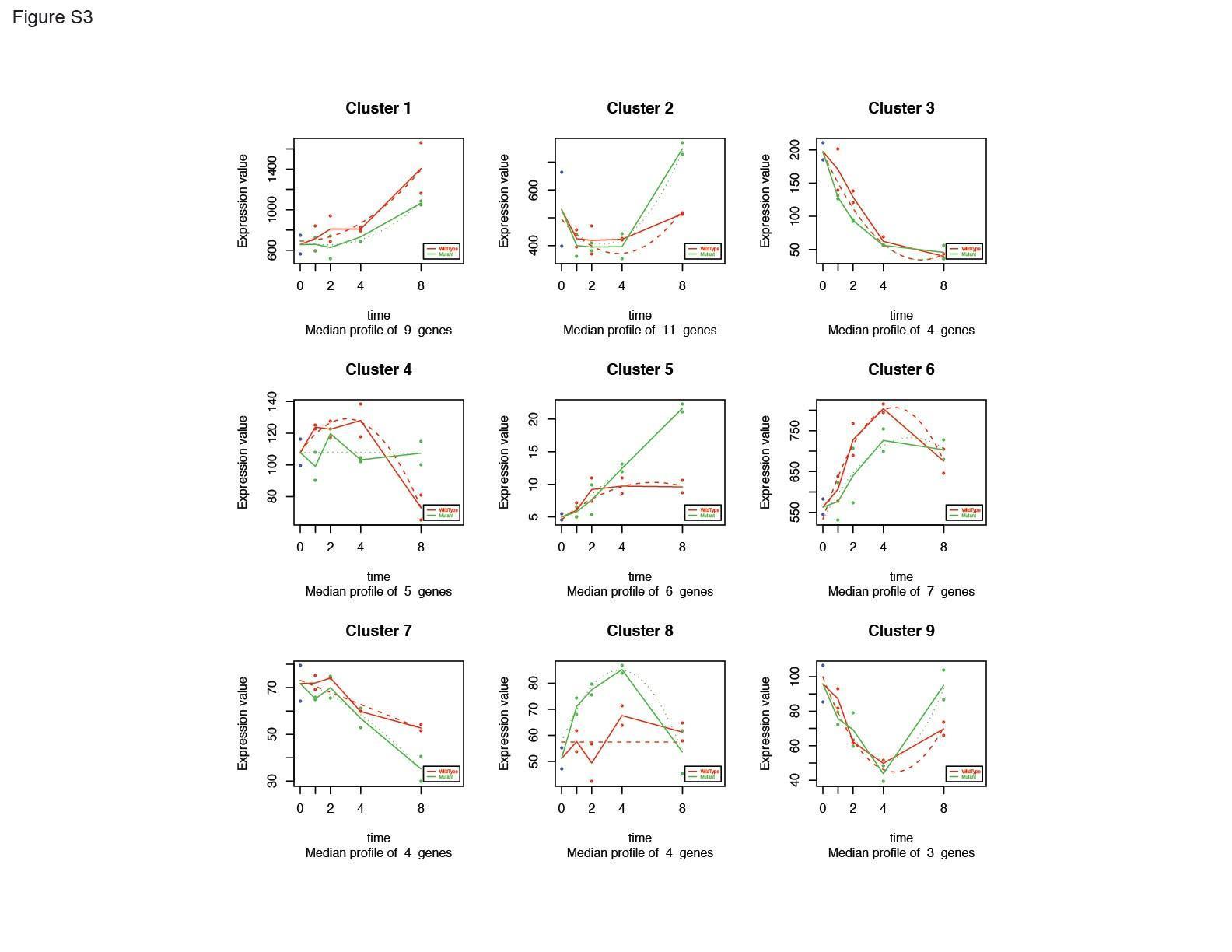


**Supplementary Figure 3. Gene expression changes in Caco-2 cells cultured in transwell with BTWT or SLMUT over an 8-hour time course.** Changes in gene expression were monitored over an 8-hour time period by RNA-seq. Time course measurements were made in duplicate. Clusters of genes with significantly different expression profiles in Caco-2 cells over the 8-hour time course between BTWT (WildType - red) and SLMUT (Mutant - green) incubated cells. The x-axis shows sequencing depth normalized read count values. Solid lines connect the average expression values within a condition over time and dotted lines are the regression fit. Categories of gene functions and gene names are included in Supplementary Table 1. Source data are provided in Additional File 2.


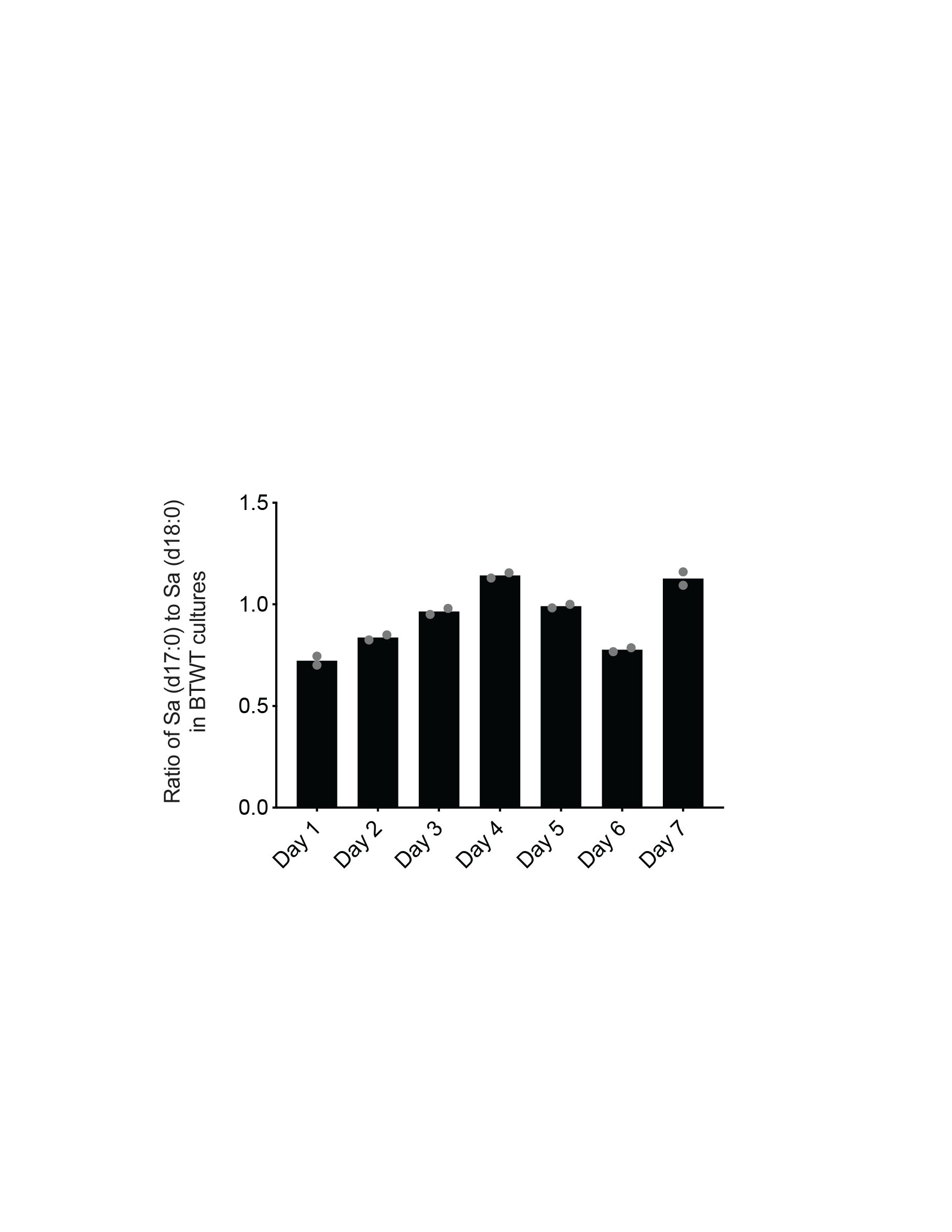


**Supplementary Figure 4. Sphinganine levels in BTWT cultures used to gavage germfree mice.** Ratio of sphinganine (Sa (d17:0)) to sphinganine (Sa (d18:0)) levels in BTWT cultures grown in minimal media as measured by LC-MS (relative response). Means ± SEM of LC-MS measurements (n=2) are plotted. Source data are provided as a source data file.


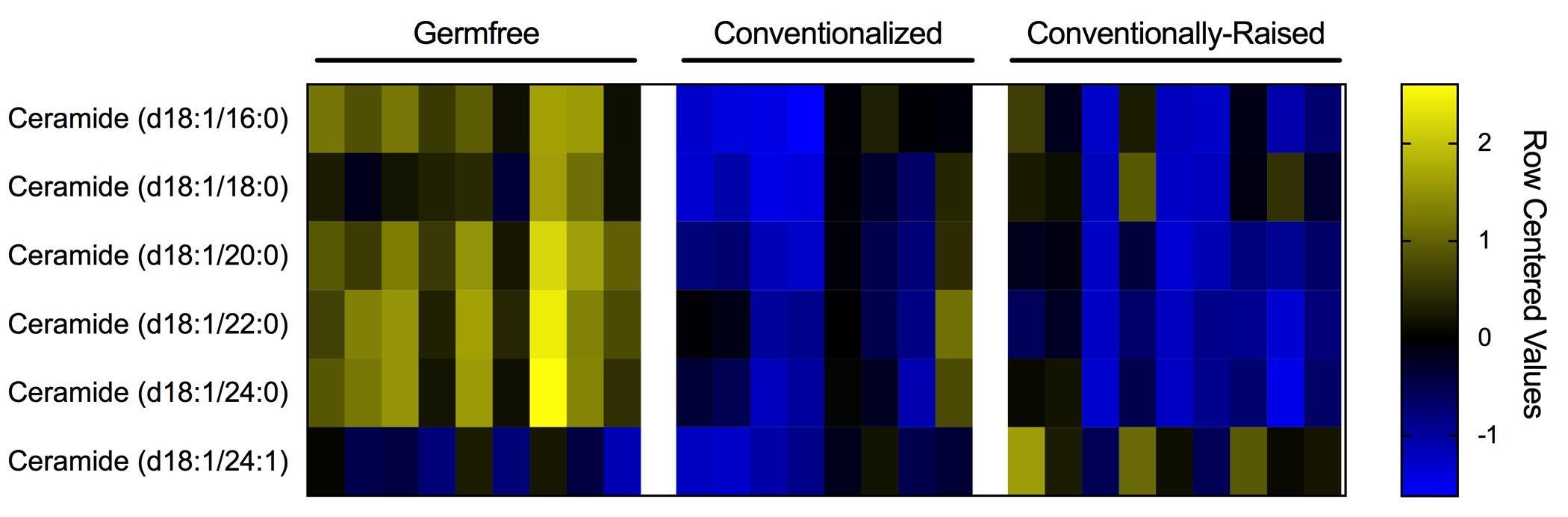


**Supplementary Figure 5. Hepatic sphingolipid levels in germfree, conventionalized, and conventionally-raised mice.** Row centered values calculated from the LC-MS determined final concentration (pmol/μg protein) of hepatic sphingolipids in germfree mice (n=9, left), germfree mice one-week after introduction of a mouse microbiota (conventionalized, n=8, middle), and conventionally-raised mice (n=9, right). Source data are provided as a source data file.

**
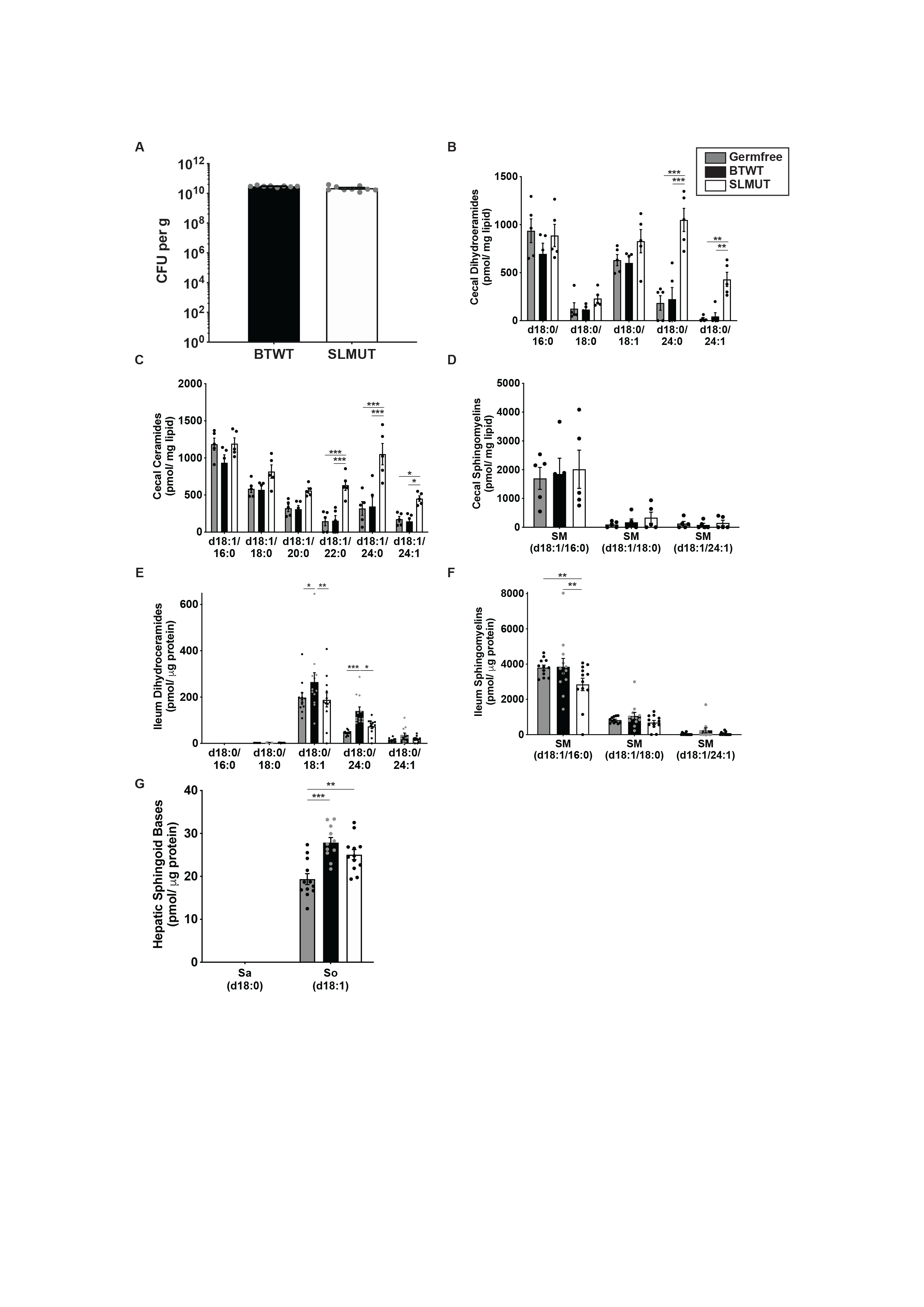
**

**Supplementary Figure 6. Colonization and sphingolipid levels of mice monoassociated with BTWT or SLMUT.** (A) Colonization efficiency of BTWT (n=8) and SLMUT (n=9) strains gavaged into germfree mice in CFU/g as measured by serial dilution of cecal content.

(B-H) Levels of SLs in different sample types obtained from: germfree mice (grey), germfree mice monoassociated with BTWT (black), and germfree mice monoassociated with SLMUT (white).

(B) Cecal dihydroceramides; (C) cecal ceramides; (D) cecal sphingomyelins. (B-D) Bar charts represent mean sphingolipid abundance ± SEM for 5 mice per condition (two-way ANOVA, Tukey’s multiple comparison test, *p<0.05, **p<0.01, ***p<0.001). (E) Ileum dihydroceramides; (F) ileum sphingomyelins; (G) hepatic long chain bases. (E-G) Bar charts represent mean sphingolipid abundance ± SEM for 12 mice per condition (germfree, SLMUT) and 11 mice per condition (BTWT), (two-way ANOVA, Tukey’s multiple comparison test, *p<0.05, **p<0.01, ***p<0.001). Source data are provided as a source data file.

**Supplementary Figure 7. Hepatic *de novo* sphingolipid synthesis is induced and C_24:1_ is the major ceramide species detected in livers of mice on a fatty acid free diet.**

(A) Ratio of hepatic dihydroceramides to total ceramides (DHcer:total Cer) in livers of mice on a fatty acid free diet as compared to mice on a breeder diet. Values are mean ± SEM and n=3 mice per condition. Observations with significant differences in sphingolipid abundance are marked with stars (two-sided t-test, *=p <0.05).

(B) Hepatic ceramide levels in mice fed a fatty acid free diet. Values are mean ± SEM and n=3 mice per condition. Source data are provided as a source data file.

**Supplementary Figure 8. Sphingolipid levels in HFD-fed mice supplemented with BTWT or SLMUT.**

Hepatic dihydroceramides (A) and sphingomyelins (B) were measured at the conclusion of the experiment. (A-B) Mean values ± SEM are plotted, n=9 BTWT; n=10 SLMUT. (A-B) Observations with significant differences in SL abundance between treatments are marked with stars (two-sided t-test, *=p <0.05). Source data are provided as a source data file.

**Supplementary Tables**

**Supplementary Table 1. Genes with significant changes in expression profiles over an 8-hour time course in Caco-2 cells incubated with BTWT or SLMUT**

Names and corresponding cluster identity of genes with significant differences in expression profiles between BTWT and SLMUT Caco-2 incubation conditions. Visualization of cluster expression profiles is shown in Supplementary Fig. 3. Protein coding genes are in black while non-coding genes are in grey. Ten pseudo/unannotated genes are not listed.

| GeneSymbol | Gene Name or Ensembl ID | Cluster |
| --- | --- | --- |
| AHSA1 | AHA1, activator of heat shock 90kDa protein ATPase homolog 1 (yeast) | 1 |
| CDCA5 | cell division cycle associated 5 | 1 |
| HMX2 | homeobox (H6 family) 2 | 1 |
| MRPL18 | mitochondrial ribosomal protein L18 | 1 |
| NEIL2 | nei like 2 (E. coli) | 1 |
| NIF3L1 | NIF3 NGG1 interacting factor 3-like 1 (S. pombe) | 1 |
| PGAM1 | phosphoglycerate mutase 1 (brain) | 1 |
| ZNF692 | zinc finger protein 692 | 1 |
| AQP11 | aquaporin 11 | 2 |
| BBS2 | Bardet-Biedl syndrome 2 | 2 |
| HIST1H2AC | histone cluster 1, H2ac | 2 |
| METTL7A | methyltransferase like 7A | 2 |
| NUDT16 | nudix (nucleoside diphosphate linked moiety X)-type motif 16 | 2 |
| PCYOX1L | prenylcysteine oxidase 1 like | 2 |
| PTPRCAP | protein tyrosine phosphatase, receptor type, C-associated protein | 2 |
| RASD1 | RAS, dexamethasone-induced 1 | 2 |
| ZFP36L2 | zinc finger protein 36, C3H type-like 2 | 2 |
| THAP7-AS1 | ENSG00000230513 | 2 |
| UBL7-AS1 | ENSG00000247240 | 2 |
| RNA Gene, and is affiliated with the lncRNA class | ENSG00000261888 | 2 |
| LINC01569 | ENSG00000262468 | 2 |
| HECA | headcase homolog (Drosophila) | 3 |
| TFDP2 | transcription factor Dp-2 (E2F dimerization partner 2) | 3 |
| RNA gene | ENSG00000224905 | 3 |
| RNA gene | ENSG00000271643 | 3 |
| C16orf72 | chromosome 16 open reading frame 72 | 4 |
| PSPC1 | paraspeckle component 1 | 4 |
| RN7SL809P | ENSG00000241217 | 4 |
| HNRNPA1P59 | ENSG00000230280 | 5 |
| RNA Gene, and is affiliated with the antisense RNA class. | ENSG00000235078 | 5 |
| HSPA14 | heat shock 70kDa protein 14 | 6 |
| RNA Gene, and is affiliated with the antisense RNA class. | ENSG00000263823 | 6 |
| ABCA12 | ATP-binding cassette, sub-family A (ABC1), member 12 | 7 |
| RNA Gene, and is affiliated with the ncRNA class | ENSG00000231999 | 7 |
| BHLHE40-AS1 | ENSG00000235831 | 7 |
| PAXBP1-AS1 | ENSG00000238197 | 7 |
| ABHD10 | abhydrolase domain containing 10 | 8 |
| EIF4A2 | eukaryotic translation initiation factor 4A, isoform 2 | 8 |
| ZNF239 | zinc finger protein 239 | 8 |
| FNTB | farnesyltransferase, CAAX box, beta | 9 |

**Supplementary Table 2. Bacterial strains and plasmids used in the generation of a BT0870 knockout strain.**

| **Strains** | | |
| --- | --- | --- |
| *BS-3234 E. coli S17-1 λpir* | Biomedal (Spain) | *recA pro hsdR RP4-2-Tc::Mu-Km::Tn7 λ-pir* |
| *B. thetaiotaomicron* VPI-5482 tdk | Andrew Goodman (Yale) | PMID_18611383 |
| **Primers** | | |
| 0870_frag1_fwd | GGTATCGATAAGCTTGATttttattgtttgctgttttggttggt | fragment 1 for 0870 deletion |
| 0870_frag1_rev | gtataataaattataattcgcagcatctctgaaaagctataaaaaagc | fragment 1 for 0870 deletion |
| 0870_frag2_fwd | gcttttcagagatgctgcgaattataatttattatactcatactcagatcattcgc | fragment 2 for 0870 deletion |
| 0870_frag2_rev | TCCACCGCGGTGGCGGCCgactccgttttccgtttccag | fragment 2 for 0870 deletion |

**Supplementary Table 3. Sphingolipids detected by LC-MS**

| **Compound Name** | **Precursor Ion** | **Product Ion** | **Fragmentation (V)** | **Collision Energy (V)** | **Ret Time (min)** | **Polarity** |
| --- | --- | --- | --- | --- | --- | --- |
| Sa (m18:0) | 288.3 | 240.2 | 119 | 24 | 1.169 | Positive |
| Cer (d18:1/12:0) | 482.46 | 464.5 | 124 | 4 | 2.751 | Positive |
| Cer (d18:1/16:0) | 538.5 | 520.5 | 119 | 4 | 3.404 | Positive |
| Cer (d18:0/16:0) | 540.5 | 522.5 | 71 | 20 | 3.545 | Positive |
| S1P (d17:1) | 366.24 | 250.2 | 99 | 8 | 1.666 | Positive |
| Sa (d17:0) | 288.29 | 270.3 | 99 | 8 | 1.046 | Positive |
| Sa1P (d17:0) | 368.24 | 252.3 | 134 | 12 | 1.72 | Positive |
| So (d17:1) | 286.3 | 268.3 | 101 | 4 | 0.954 | Positive |
| Cer (d18:1/18:0) | 566.6 | 264.3 | 139 | 28 | 3.866 | Positive |
| Cer (d18:0/18:0) | 568.6 | 550.6 | 172 | 20 | 4.09 | Positive |
| Cer (d18:0/18:1) | 566.6 | 548.54 | 172 | 16 | 4.134 | Positive |
| Cer (d18:1/20:0) | 594.6 | 264.3 | 129 | 24 | 4.534 | Positive |
| Cer (d18:1/22:0) | 622.6 | 264.3 | 167 | 20 | 5.4 | Positive |
| Cer (d18:1/24:0) | 650.7 | 264.3 | 114 | 28 | 6.7 | Positive |
| Cer (d18:0/24:0) | 652.7 | 634.6 | 205 | 16 | 6.5 | Positive |
| Cer (d18:1/24:1) | 648.6 | 264.3 | 99 | 24 | 5.35 | Positive |
| Cer (d18:0/24:1) | 650.7 | 632.6 | 157 | 20 | 6.2 | Positive |
| S1P (d18:1) | 380.3 | 264.3 | 129 | 12 | 1.87 | Positive |
| Sa (d18:0) | 302.3 | 284.3 | 109 | 8 | 1.75 | Positive |
| Sa1P (d18:0) | 382.3 | 284.3 | 119 | 8 | 1.92 | Positive |
| SM (18:1/16:0) | 703.6 | 184.1 | 195 | 28 | 3.12 | Positive |
| SM (18:1/18:0) | 731.6 | 184.1 | 215 | 28 | 3.55 | Positive |
| SM (18:1/18:1) | 729.6 | 184.1 | 195 | 28 | 3.15 | Positive |
| SM (18:1/24:0) | 815.7 | 184.1 | 220 | 28 | 5 | Positive |
| SM (18:1/24:1) | 813.7 | 184.1 | 215 | 28 | 4.7 | Positive |
| So (d18:1) | 300.3 | 282.3 | 101 | 4 | 1.69 | Positive |
