# Supplementary material for: Sphingolipids produced by gut bacteria enter host metabolic pathways impacting ceramide levels
Source: Nat Commun. 2020 May 18;11:2471. doi: 10.1038/s41467-020-16274-w (PMC7235224; doi:10.1038/s41467-020-16274-w)
Supplement: Supplementary file 2 — Description of Additional Supplementary Files [file 41467_2020_16274_MOESM2_ESM.docx]

**Description of Additional Supplementary Files**

File Name: Supplementary Data 1

Description: Excel file containing SL genes used in RNA-seq analysis

File Name: Supplementary Data 2

Description: Excel file containing RNA-seq read counts
