# Supplementary material for: Sphingolipids produced by gut bacteria enter host metabolic pathways impacting ceramide levels
Source: Nat Commun. 2020 May 18;11:2471. doi: 10.1038/s41467-020-16274-w (PMC7235224; doi:10.1038/s41467-020-16274-w)
Supplement: Supplementary file 5 — Reporting Summary [file 41467_2020_16274_MOESM5_ESM.pdf]

nature research

Corresponding author(s): Ruth Ley

Last updated by author(s): Apr 3, 2020

Reporting Summary

Nature Research wishes to improve the reproducibility of the work that we publish. This form provides structure for consistency and transparency in reporting. For further information on Nature Research policies, see Authors & Referees and the Editorial Policy Checklist.

Statistics

For all statistical analyses, confirm that the following items are present in the figure legend, table legend, main text, or Methods section.

n/a	Confirmed	
☐	☒	The exact sample size (n) for each experimental group/condition, given as a discrete number and unit of measurement
☐	☒	A statement on whether measurements were taken from distinct samples or whether the same sample was measured repeatedly
☐	☒	The statistical test(s) used AND whether they are one- or two-sided<br>*Only common tests should be described solely by name; describe more complex techniques in the Methods section.*
☒	☐	A description of all covariates tested
☒	☐	A description of any assumptions or corrections, such as tests of normality and adjustment for multiple comparisons
☐	☒	A full description of the statistical parameters including central tendency (e.g. means) or other basic estimates (e.g. regression coefficient) AND variation (e.g. standard deviation) or associated estimates of uncertainty (e.g. confidence intervals)
☐	☒	For null hypothesis testing, the test statistic (e.g. F, t, r) with confidence intervals, effect sizes, degrees of freedom and P value noted<br>*Give P values as exact values whenever suitable.*
☒	☐	For Bayesian analysis, information on the choice of priors and Markov chain Monte Carlo settings
☒	☐	For hierarchical and complex designs, identification of the appropriate level for tests and full reporting of outcomes
☒	☐	Estimates of effect sizes (e.g. Cohen's d, Pearson's r), indicating how they were calculated

Our web collection on statistics for biologists contains articles on many of the points above.

Software and code

Policy information about availability of computer code

Data collection	We used Mass Hunter Workstation Software Version B.06.00 SP01/Build 6.0.388.1 for collection of sphingolipid LC-MS data. We used Zeiss Zen 2012 SP (black edition).
Data analysis	GraphPad Prism 8.3.0, R 3.3.2, R packages maSigPro, nlme, multcomp, MassHunter Workstation Software Version 8.06.00 SP01/Build6.0.388/1, Image J v1.51

For manuscripts utilizing custom algorithms or software that are central to the research but not yet described in published literature, software must be made available to editors/reviewers. We strongly encourage code deposition in a community repository (e.g. GitHub). See the Nature Research guidelines for submitting code & software for further information.

Data

Policy information about availability of data

All manuscripts must include a data availability statement. This statement should provide the following information, where applicable:

- Accession codes, unique identifiers, or web links for publicly available datasets
- A list of figures that have associated raw data
- A description of any restrictions on data availability

Provide your data availability statement here.

Field-specific reporting

Please select the one below that is the best fit for your research. If you are not sure, read the appropriate sections before making your selection.

☒ Life sciences ☐ Behavioural & social sciences ☐ Ecological, evolutionary & environmental sciences

Life sciences study design

All studies must disclose on these points even when the disclosure is negative.

Sample size	Sample sizes were calculated based on sphingolipid measurements done in germ-free mice where n= 10 was sufficient to detect difference in sphingolipid abundance between the GF and conventionalized state in liver, serum, and ileum tissue. Sample sizes to measure dhCer to Cer ratios in FAF diet mice were determined from pilot experiments where n=4 mice per condition were sufficient to detect significant differences in the dhCer to Cer ration between treatment conditions. Replicates for tissue culture experiments (biological replicates n= 3) were determined by reviewer input. No predermined methods were used to generate sample sizes for hepatic portal vein or alkyne-labeled bacterial lipid uptake experiments. Sample sizes for uptake experiments in mice were deemed sufficient as uptake was constantly observed over time, controls were negative for uptake and replication attempts (see below) were successful.
Data exclusions	No data were excluded
Replication	Cell culture experiments were completed in three separate biological experiments except the gene expression data which was measured in duplicate. Measurements of sphingolipid abundances in replicates of mouse experiments in germ-free mice and mice fed a fatty acid free diet were successful. Lipid uptake experiments in mice have been successfully repeated as routine controls for ongoing experiments. All attempts at replication were successful.
Randomization	Randomization of cell culture was not needed as all cells came from the same source. Randomization of mice were not needed as mice within each experiment came from the same source and had similar weight distributions between treatments.
Blinding	In this study, the measurements were not subjective and blinding was not necessary.

Reporting for specific materials, systems and methods

We require information from authors about some types of materials, experimental systems and methods used in many studies. Here, indicate whether each material, system or method listed is relevant to your study. If you are not sure if a list item applies to your research, read the appropriate section before selecting a response.

Materials & experimental systems

n/a	Involved in the study	
☒	☐	Antibodies
☐	☒	Eukaryotic cell lines
☒	☐	Palaeontology
☐	☒	Animals and other organisms
☒	☐	Human research participants
☒	☐	Clinical data

Methods

n/a	Involved in the study	
☒	☐	ChIP-seq
☒	☐	Flow cytometry
☒	☐	MRI-based neuroimaging

Eukaryotic cell lines

Policy information about cell lines

Cell line source(s)	Caco-2 Human Colon Epithelial Cells (ATCC)
Authentication	None of the cell lines used were authenticated
Mycoplasma contamination	Cell lines were not tested for mycoplasma contamination
Commonly misidentified lines (See ICLAC register)	No commonly misidentified cell lines used

Animals and other organisms

Policy information about studies involving animals; ARRIVE guidelines recommended for reporting animal research

Laboratory animals	Mus musculus, Swiss Webster, Female Mice, 3-6 weeks of age
Wild animals	Not used
Field-collected samples	Not used
Ethics oversight	Cornell University Institutional Animal Care and Use Committee, Protocol #2010-0065

Note that full information on the approval of the study protocol must also be provided in the manuscript.
